# Macrophage heterogeneity and its interactions with stromal cells in tumour microenvironment

**DOI:** 10.1186/s13578-024-01201-z

**Published:** 2024-02-01

**Authors:** Liren Cao, Xiaoyan Meng, Zhiyuan Zhang, Zhonglong Liu, Yue He

**Affiliations:** Department of Oral Maxillofacial & Head and Neck Oncology, National Clinical Research Center for Oral Disease, National Center of Stomatology, Shanghai Ninth People’s Hospital Affiliated to Shanghai Jiao Tong University School of Medicine, Shanghai, 200011 China

**Keywords:** Single-cell study, Tumour-associated macrophage, Macrophage heterogeneity, Tumour stroma, Macrophage–stroma interactions

## Abstract

Macrophages and tumour stroma cells account for the main cellular components in the tumour microenvironment (TME). Current advancements in single-cell analysis have revolutionized our understanding of macrophage diversity and macrophage–stroma interactions. Accordingly, this review describes new insight into tumour-associated macrophage (TAM) heterogeneity in terms of tumour type, phenotype, metabolism, and spatial distribution and presents the association between these factors and TAM functional states. Meanwhile, we focus on the immunomodulatory feature of TAMs and highlight the tumour-promoting effect of macrophage–tumour stroma interactions in the immunosuppressive TME. Finally, we summarize recent studies investigating macrophage-targeted therapy and discuss their therapeutic potential in improving immunotherapy by alleviating immunosuppression.

## Introduction

The tumour microenvironment (TME) is defined as a complex multicellular system typically comprising immune cells, stromal cells, extracellular matrix (ECM), and various secreted factors, in which these noncancerous components communicate with each other to facilitate tumour development. Among non-cancerous cell components, immune cells, in particular tumour-associated macrophages (TAMs), have attracted researchers’ attention since the mid-nineteenth century due to their accelerating roles in tumour progression [1]. It is well known that TAMs are capable of promoting tumour cell survival, invasion, and metastasis [2]. TAMs are regarded as the main immunomodulatory cells in the TME and can directly suppress the activation of T lymphocytes and natural killer (NK) cells to compromise the protective immune response [3, 4]. Tumour stromal cells are mainly composed of but not limited to cancer-associated fibroblasts (CAFs), endothelial cells (ECs), pericytes, and mesenchymal stem cells (MSCs). Among them, CAFs play important roles in multiple biological process during disease progression, including carcinogenesis, angiogenesis, and immunosuppression [5–7]. Notably, TAM–CAF interactions regulate the tumour immune microenvironment and accelerate tumour progression. A previous review has presented the complicated TAM–CAF crosstalk network [8]. Although other stromal cells are not the main immunomodulatory cells in the TME, an increasing number of studies have discovered that communication between macrophages and these cells prompts immune suppression and tumour development [9]. Thus, we think TAMs should be the key regulatory cells in TME that facilitate tumour progression through complex cell communications. How TAMs interact with these stromal cells? And what effect would be triggered? Clearly understanding these cellular crosstalk mechanisms may boost new cancer therapy. However, to the best of our knowledge, no review has systematically delineated the interaction networks between TAMs and tumour stromal cells.

**Fig. 1:**
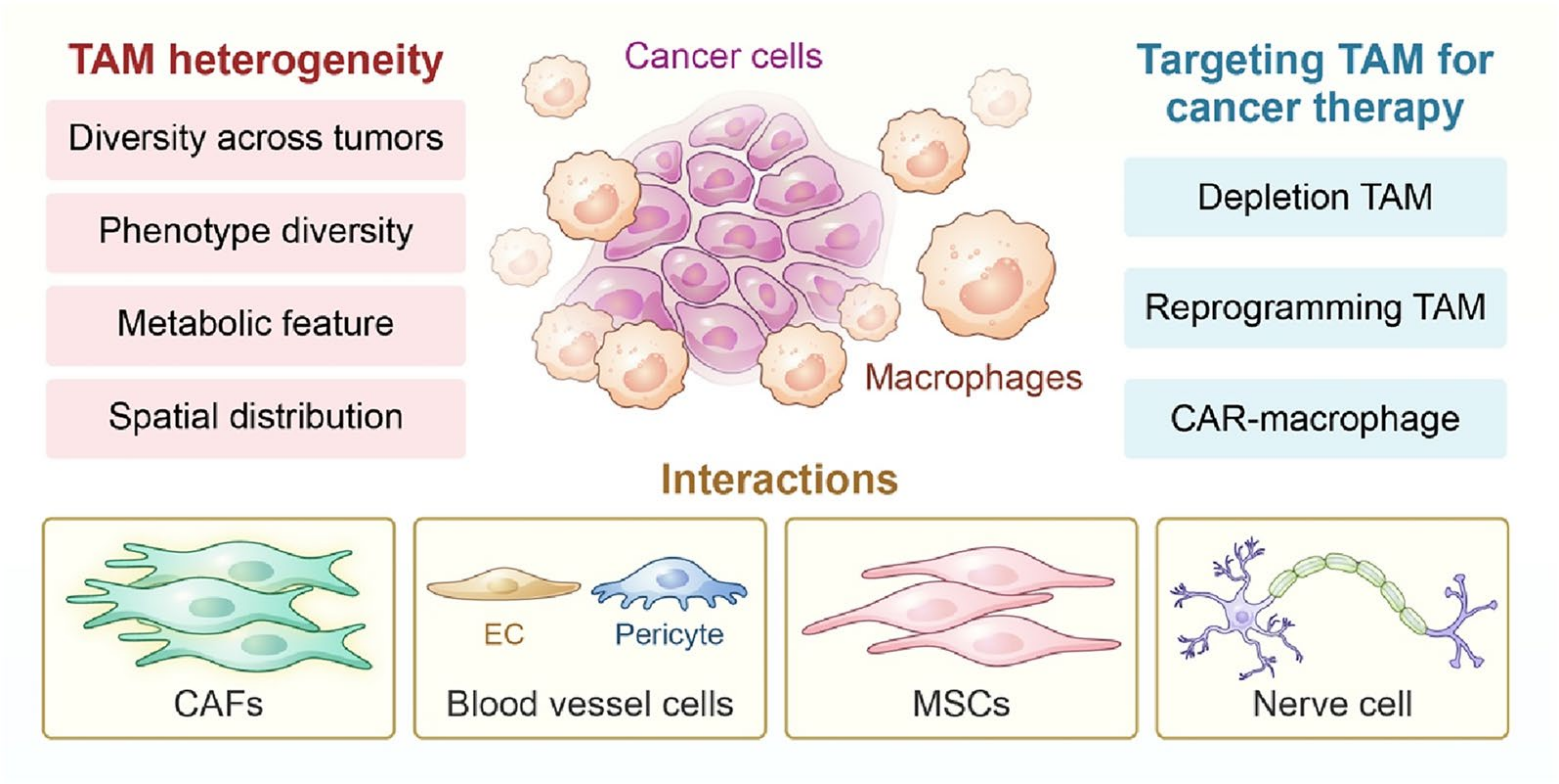
Schematic illustration of main contents in this review. (1) TAM heterogeneity in four aspects: tumour type, phenotype, metabolism, and spatial distribution; (2) TAMs interact with stromal cells, including CAFs, ECs, pericytes, MSCs, and nerve cells, in tumour microenvironment; (3) Current main strategy for TAM-targeted therapy

Currently, single-cell studies provide a deeper understanding of macrophages and their interactions with tumour stromal cells. Thus, based on new insights from these studies, in this review, we aimed to describe the heterogeneity of TAMs as comprehensive as possible. And then depiction of the complex crosstalk networks between TAMs and other important stromal components were presented, including CAFs, ECs, pericytes, and MSCs. Finally, we introduced existing TAM-targeted therapeutic strategies for relieving immunosuppression in the TME and enhancing immunotherapy (as showed in Fig. 1).

## Single-cell studies reveal heterogeneity of TAMs

Macrophages are recognized as key stromal components in tumours, profoundly affecting the composition and characteristics of the TME. Macrophages have long been considered to have two distinct populations: (1) proinflammatory macrophages, induced by Toll-like receptor (TLR), interferon (IFN)-γ and tumour necrosis factor (TNF)-α, are called M1 macrophages; and (2) anti-inflammatory macrophages, activated by interleukin (IL)-4 or IL-13, are termed M2 macrophages. M1-type TAMs are usually involved in activating antitumour immunity and can produce nitric oxide (NO), reactive oxide species (ROS), and multiple proinflammatory cytokines, such as TNFα, IL-1β, and IL-6 [10]. In contrast, M2-type TAMs are pro-tumour subpopulations that are responsible for tumour progression and poor clinical outcomes [11–13]. Although the M1/M2 phenotypes sketch the different functional states of macrophages, this traditional classification remains oversimplified. Classical M2 macrophages are not single cell populations but consist of different phenotypic subsets with diverse functions and cell surface marker expression [10, 14]. Moreover, some mixed-polarized TAMs co-express both M1 and M2 gene signatures [15, 16]. Owing to the development of single-cell RNA sequencing (scRNA-seq), our understanding of macrophage polymorphisms has been revolutionized, which more accurately describes the phenotypic and functional diversity of macrophages at an in-depth molecular signature level. Based on single-cell data, TAMs can be divided into various subsets according to their gene signature and enriched pathway or key cell surface markers. Evidence is emerging that TAM diversity strongly depends on tumour type. TAMs identified in different tumour types may be characterized by specific markers. Significantly, metabolism is another layer of macrophage heterogeneity that is highly correlated with their phenotypic diversity. Metabolic pathways in macrophages switch during polarization, along with phenotypic and functional changes [17]. It is well known that the metabolic features of macrophages regulate their gene expression and functions as well [18, 19]. Furthermore, spatial transcriptome data revealed that macrophages located in different regions within tumours exhibit dissimilar functions. This difference might be attributed to cell interactions between TAMs and other tumour stromal cells. Collectively, new insight from single-cell studies reveals that the heterogeneity of TAMs underlies cancer type, phenotype, metabolism, and spatial distribution (summarized in Table 1).

**Table 1:** Heterogeneity of TAMs in the tumour microenvironment

TAM	Tumour	Spatial distribution	Metabolic features	Property and function	References
APOE^+^CTSZ^+^ TAMs	CRC	Colocalize with Treg cells	Enhanced glutamine metabolism	Immunosuppression function	[65]
Lipid-associated macrophages (LAMs)	BC; HCC	Tumour-adipose adjacent regions; Tumour invasion regions	Upregulation of lipid metabolism	Extracellular matrix remodelling; recruit Treg cell; inhibit T-cell activation	[35–37]
C1Q^+^TREM2^+^APOE^+^ macrophages	ccRCC	–	–	Associate with postsurgical recurrence	[28]
TREM2^+^ macrophages	NSCLC	–	Upregulation of lipid metabolism	Interaction with Treg; suppress CD8^+^ T-cell function	[38]
MARCO^+^ macrophages	GBM; NSCLC; MEL	Perivascular areas	Anti-MARCO treatment increases glycolysis in MEL	Downregulate inflammatory pathways; weak antigen presentation capability; suppress T-cell an NK cell function; support tumour vascularization	[39–41, 43]
MMP9^+^ macrophages	HCC; Gastric cancer; PDAC	–	–	Terminal differentiated TAMs; promote tumour cell invasion and migration; angiogenesis; promote epithalami–mesenchymal transition	[44–46]
FLOR2^+^ macrophages	BC	Perivascular niche	–	Trigger CD8^+^ T-cell activation	[47]
^TLS^TIM4^+^FOLR2^+^ macrophages	LCA; SCC; CRC; Gastric cancer	Tertiary lymphoid structures	–	Correlate to active immune infiltration and antitumour immune response	[25]
embryonic-like FOLR2^+^ macrophages	HCC	Perivascular niche	–	Express high level immunomodulatory chemokines; interact with Tregs	[48]
CD163^+^TIM4^+^ omental macrophages	Ovarian cancer	–	–	Promote cancer stem cell-like properties acquisition and epithalami–mesenchymal transition	[49]
TUBB3^+^ macrophages	LCA	–	–	Neuron-like cell transition; exhibit nociceptor-like activities	[31]
IL2RA^+^ VSIG4^+^ macrophages	ATC	–	–	Bifunction phenotype: promote immunosuppression; associate with increased lymphocytes (B cells, CD8+ T cells, Tregs) infiltration	[50]
LAM2: APOE/c2	BC	Tumour invasive regions	High expression of lipid metabolism genes	Immunosuppressive function	[72]
F4/80^+^ TAMs	CRC liver metastasis	Perivascular areas	–	Angiogenic function	[73]
Lyve-1^+^ TAMs	BC	Perivascular areas	–	Direct αSMA^+^ CAFs expansion to form pro-angiogenesis niche	[74]
PDPN^+^ TAMs	BC	Perilymphatic localization	–	Support lymphangiogenesis and tumour lymph-invasion Extracellular matrix remodelling	[32]
SPP1^+^ TAMs	HCC	Tumour border proximal to CAFs	High expression of lipid metabolism genes	Interact with CAFs to form immunosuppressive TME	[75]
MRC1^+^ TAMs	BC	Perivascular areas	–	Angiogenic function	[77]
CD169^+^ lymph node macrophages	BC metastasis	Tumour-draining lymph node	–	Anti-metastasis function	[81]

*BC* breast cancer, *HCC* hepatocellular carcinoma, *ccRCC* clear cell renal carcinoma, *NSCLC* non-small cell lung cancer, *GBM* glioblastoma, *MEL* melanoma, *PDAC* pancreatic ductal adenocarcinoma, *LCA* lung cancer, *SCC* squamous cell carcinoma, *CRC* colorectal cancer, *ATC* anaplastic thyroid cancer

### TAM diversity across tumours

The organ environment determines the composition of TAM subpopulations. Lehmann et al. discovered that TAMs showed distinct TAM populations in a mouse melanoma model in the skin or lung, in which blood-derived TAMs were predominant populations in skin tumours and responsible for the antibody-dependent immunotherapy response, whereas tissue-resident alveolar macrophages were the main effector cells in lung tumours [20]. TAMs originate from tissue-resident macrophages (TRMs) or newly recruited monocytes and are reprogrammed by tumour cells or stromal cells to support tumour progression. In some specific tumours, TRMs are an important source of TAMs, which are critical for tumorigenesis. In pancreatic cancer, TAMs originating from embryonically derived TRMs undergo expansion during tumour progression and exhibit a profibrotic phenotype [21]. Moreover, in lung tumours, alveolar macrophages, showing distinct transcripts from monocyte-derived macrophages, provide a pro-tumorigenic niche at the early stage of tumour progression [22]. These data imply that different origins of TAMs may contribute to subpopulation diversity across tumours.

With the application of the scRNA-seq technique, TAMs can be classified into more precise subsets according to surface markers. This phenotypic diversity of macrophages across several of the most common tumours has been reviewed recently [23]. Some TAM markers are universally identified in various tumours, while some are restricted to specific types. For example, C1q, TREM2, and FOLR2 are all key biomarkers for identifying TAM subsets in various cancers [24–26]. According to recent single-cell studies, C1q^+^ TAMs were detected in multiple malignant tumours, including hepatocellular carcinoma (HCC), clear cell renal cell carcinoma (ccRCC), pancreatic ductal adenocarcinoma (PDAC), and colorectal cancer, and are associated with immune suppression and poor prognosis [27–30]. These results suggest that C1q^+^ TAMs are pro-tumour macrophage subpopulations that extensively exist in various tumours, and C1q can be used as a marker of poor clinical outcomes. Similar TAM subsets include MPP9^+^ TAMs, MACRO^+^ TAMs, and lipid-associated macrophages, all of which are extensively detected in a multitude of tumours (Table 1). However, several TAM subsets have only been discovered in specific tumours. Tang et al. reported a neuron-like macrophage subset, TUBB3^+^ TAMs, in lung adenocarcinoma with the property of promoting tumour neurogenesis [31]. In addition, podoplanin-expressing macrophages (PDPN^+^ TAMs) are another tumour-specific macrophage subset that has only been reported in breast cancer [32]. These PDPN^+^ TAMs, localized proximally to lymphatic vessels, could migrate and adhere to lymphatic endothelial cells depending on podoplanin-galectin 8 binding and then integrin β1 activation. This cell-to-cell contact would support lymphangiogenesis by sustaining lymphatic vessel sprouting.

### Phenotypic diversity and functional plasticity of TAMs

As mentioned before, TAMs present phenotypic heterogeneity. Diverse TAM subgroups were recently discovered in various types of cancers. Lipid-associated macrophages (LAMs) were found in adipose tissue but were also isolated from mouse lung metastasis lesions of breast cancer [33]. This subset is characterized by enrichment for genes implicated in pathways related to lipid metabolism, extracellular matrix remodelling, immunosuppression, and epithelial–mesenchymal transition, indicating its pro-tumorigenic properties. Previous research revealed that TREM2, a kind of lipid receptor, was essential for LAM protective function to control metabolic haemostasis in adipose tissues [34]. TREM2^+^ LAMs were also detected in hepatocellular carcinoma and exhibited an immunosuppressive function by recruiting suppressive Treg cells via the CCL20/CXCL9/CXCL10/CXCL12–CXCR3 axes, which may compromise the antitumour response [35]. Additionally, LAMs induced by FAP^+^ cancer-associated fibroblasts inhibited T-cell proliferation and effector functions by secreting Granzyme-B and IL-10 [36]. It has been demonstrated that depletion of TREM2^+^ LAMs in the tumour microenvironment can potentiate the efficacy of anti-PD1 therapy in a mouse breast cancer model [37]. All this evidence suggests that the role of LAMs in immunosuppressive TME formation is crucial and that targeting LAMs may be a promising approach for immunotherapy. In other TAM subpopulations, TREM2 is also a key biomarker. A TREM2^+^ TAM subset was confirmed to accumulate in human non-small cell lung cancer, which significantly increased the expression of apolipoprotein genes (APOC1, APOC2 and APOE) and may mediate CD8^+^ T-cell exhaustion via interaction with FOXP3^+^ regulatory T lymphocytes (Tregs) to upregulate TGF-β expression [38]. In clear cell renal cancer, C1Q^+^TREM2^+^APOE^+^ TAMs were associated with cancer recurrence [28]. The scavenger receptor MARCO, normally found on alveolar macrophages, is also detected on TAMs in several cancers and defines a distinct TAM subset that has been validated to display an immunomodulatory role. At the transcriptomic level, MACRO expression positively correlated with genes linked to Tregs (FOXP3, TGFB1, IL10, CTLA4), exhausted T cells (PDCD1, TIGIT, BTLA, HAVCR2 and LAG3), and immune checkpoints comprising PD-L1, PD-1, VISTA, and CTLA4 [39]. MARCO^+^ TAMs demonstrated loss of inflammatory pathways, including the IFN-α response, IFN-γ response, allograft rejection and TNF-α signalling via NFKB, while the competence of antigen processing and presentation via MHC-II was weakened [40]. In an in vitro test, MARCO^+^ macrophages inhibited T-cell proliferation, IFN-γ production and cytotoxicity and suppressed NK cell activation [41]. These results suggest that MARCO^+^ TAMs may be a potent therapeutic target, and a previous study has proven that targeting MARCO could inhibit tumour growth and metastasis [42]. Recently, research revealed that MARCO^+^ TAMs exhibit a perivascular macrophage phenotype and that targeting MARCO-expressing macrophages can suppress the capability of MARCO^+^ TAMs to support tumour vascularization and activate natural killer (NK) cell killing via TNF-related apoptosis ligands only [43]. MMP9^+^ TAMs are another crucial subset in the TME, correlating to the epithalami–mesenchymal transition (EMT) of tumour cells. Lu et al. identified MMP9^+^ TAMs, a terminal differentiated subpopulation accumulated from TREM2^+^ TAMs and monocyte-derived macrophages, and PPARγ was a driving molecule of differentiation and subsequently promoted HCC progression by inducing HCC cell migration, invasion, and tumour angiogenesis [44]. It has been demonstrated that activation of PI3K/AKT by matrix metalloprotease 9 (MMP9) can induce the PI3K/AKT downstream transcription factor Snail to facilitate EMT via vimentin upregulation and E-cadherin downregulation, resulting in enhanced tumour invasion and metastasis [45]. Moreover, MMP9^+^ TAMs induced EMT in pancreatic cancer by secreting MMP9 to activate protease-activated receptor 1 (PAR1), which allowed tumour cells to escape macrophage-dependent cell death [46]. Interestingly, although most TAMs are typically considered immunosuppressive and pro-tumorigenic, some research has identified special macrophage subsets with a protective role in the TME. Nalio et al. demonstrated that FOLR2^+^ tissue-resident macrophages, present in both healthy mammary glands and breast tumours, localized in perivascular areas in the tumour stroma and responded to tumour progression by triggering CD8^+^ T-cell activation to enhance antitumour immunity [47]. Similarly, a TIM4^+^FOLR2^+^ macrophage subpopulation was demonstrated to localize in tertiary lymphoid structures and correlate with active immune infiltration and the antitumour immune response [25]. Notably, macrophages expressing TIM4 and FOLR2 can also predict a poor prognosis of patients. FOLR2^+^ TAMs contribute to creating an immunosuppressive microenvironment by expressing high levels of immunomodulatory chemokines (such as CXCL12, CXCL16, and CD86) and interacting with Tregs [48]. TIM4^+^ omental macrophages promoted cancer stem cell-like property acquisition and epithalami–mesenchymal transition in ovarian cancer [49] and abundantly expressed IL-10 and TGF-β [25]. In particular, IL2RA^+^VSIG4^+^ macrophages, a subtype that simultaneously expresses M1 and M2 markers, identified in anaplastic thyroid cancer were demonstrated to exhibit a bifunctional phenotype [50]. On the one hand, IL2RA^+^ VSIG4^+^ macrophages were linked to high lymphocyte infiltration, including B cells and CD8^+^ T-cells; on the other hand, they highly expressed the immune checkpoints VSIG4, LAIR1, CD86, and LILRB2, all of which are related to immunosuppression.

### Metabolic reprogramming and heterogeneity of TAMs

The metabolic profiles of TAMs are relevant to their phenotypes, and RNA-Seq analyses revealed that TAM metabolism is highly heterogeneous, and their metabolism pattern evolves over tumour development, converting towards a pro-tumorigenic pattern [51]. This evidence signifies that the TAM metabolic profile is dynamic and that the metabolic pattern is pivotal in regulating TMA phenotype transformation, eventually contributing to disease progression.

The Warburg effect, also termed aerobic glycolysis, is recognized as a hallmark of tumours. Concretely, the Warburg effect is a metabolism in which tumour cells preferentially produce lactate through glycolysis under normoxic conditions, as discovered by Warburg [52]. Subsequently, aerobic glycolysis was also observed in TAMs and correlated with their functional states [53, 54]. Geeraerts et al. found that TAMs expressing low MHC-II could secrete lactate through aerobic glycolysis, not only promoting their energy metabolism by utilizing lactate as a carbon source to fuel the tricarboxylic acid cycle but also enhancing their T-cell suppressive capacity [54]. This metabolic pattern of TAMs is stimulated by the tumour microenvironment. Proteomic analyses have revealed that the upregulation of TAM aerobic glycolysis was induced by tumour cells through a hexokinase-2-dependent pathway [55]. Moreover, tumour cell-derived microRNA was proven to downregulate lactate dehydrogenase B in macrophages to increase glycolysis [56]. Actually, the complex crosstalk network between tumour cells and macrophages accounts for the metabolic reprogramming in TAMs, and this transformation is beneficial to tumour growth. Aerobic glycolysis is crucial for TAMs to maintain an immunosuppressive phenotype and tumorigenic function. Inhibiting aerobic glycolysis in TAMs was demonstrated to significantly decrease the expression of the M2 markers CD206, CD301, and CD163 [57]. Through inhibiting the key mediator hexokinase-2, downregulation of glycolysis in TAMs resulted in attenuated competence of angiogenesis, extravasation, and epithalami–mesenchymal transition [58]. Arts et al. also demonstrated that TAM metabolism rewiring from oxidative phosphorylation towards aerobic glycolysis led to an inflammatory phenotype with both M1 and M2 markers and increased production of cytokines such as TNF and IL-6, ultimately supporting tumour progression [59]. Thus, the metabolic reprogramming in TAMs induced by tumour cells can impact the macrophage phenotype, which may contribute to pro-tumour microenvironment formation.

A similar phenomenon has also been observed in other metabolic pathways. Upregulation of genes linked to lipid metabolism was found in different immunosuppressive TAM subsets, such as TREM2^+^ macrophages, SPP1^+^ macrophages, and lipid-associated macrophages [37, 38, 60]. Long-chain fatty acid metabolism was demonstrated to facilitate the polarization of myeloid cells, expressing TAM markers such as MMP9 and VEGFα and M2 markers such as Retinal, Arg1, and CD206 [61]. It has been shown that scavenger receptor CD36 on macrophages plays a crucial role in long-chain fatty acid metabolism in the TME. Yang et al. found that CD36 expression on macrophages was upregulated in response to the TME, and then macrophages underwent M2 tumour-promoting phenotype conversion after engulfing tumour-derived lipids via a CD36-dependent mechanism [62]. The accumulated lipids in TAMs supply energy via fatty acid oxidation, and this metabolic process controls macrophage differentiation and is required for the promotion of tumour cell proliferation [63]. Additionally, lipid accumulation in TAMs caused by monoglyceride lipase deficiency induced macrophage activation towards the M2-like phenotype and suppressed CD8^+^ T cell function [64].

In addition to glucose and lipid metabolism, TAMs also exhibit elevated consumption of glutamine and arginine. Single-cell analyses revealed that a TAM subpopulation, APOE^+^CTSZ^+^ TAMs, was characterized by enhanced expression of glutamine synthetase, and the upregulation of glutamine metabolism was positively correlated with the anti-inflammatory and immunosuppressive properties of macrophages [65]. Accordingly, inhibiting glutamine metabolism showed an antitumour macrophage phenotype shift, including increased expression of TNF, TLR4, CD80 and CD86 but decreased expression of IL-10 [66]. Intriguingly, the activation of CD40 signalling promoted fatty acid oxidation and glutamine metabolism and triggered glutamine-to-lactate conversion; however, this metabolic reprogramming towards reinforced fatty acid oxidation and glutamine metabolism induced the repolarization of TAMs towards a proinflammatory and antitumorigenic phenotype [67]. The above contradictory evidence suggests that the metabolic profiles of TAMs are highly heterogeneous and dynamic, and metabolic rewiring may imply phenotypic and functional conversion in TAMs. Previous research has demonstrated that arginine metabolism of TMAs correlates with T-cell suppression [68, 69]. Recent studies found that TAMs could induce a profibrotic phenotype in pancreatic stellate cells, leading to enhanced collagen deposition in the TME through reactive nitrogen species generated in macrophage arginine metabolism, eventually promoting fibrosis in pancreatic cancer [70]. Overall, metabolic heterogeneity is a feature of TAMs, and TAM metabolism strongly affects their ability to suppress the antitumour immune response and promote tumour growth.

### Spatial heterogeneity of TAMs

Spatial heterogeneity is a hallmark of TAMs in multiple cancers and reflects their functional diversity. In breast cancer, TAMs localized in the stroma or neoplastic epithelium of the mammary duct follow distinct differentiation paths, showing specific transcriptomic signatures [71]. This evidence suggests that environmental perturbations are a key driver of TAM diversity. Wu et al. identified two LAM-like macrophage subsets in tumour invasive regions, termed LAM1: FABP5/c1 and LAM2: APOE/c2. LAM2 cells, juxtaposed with CD4^+^/CD8^+^ cells, expressed PD-L1 and PD-L2, displaying immunosuppressive functions [72]. It has been proven that TAMs in the perivascular niche are related to angiogenesis. A TAM population, F4/80^+^ macrophages, transformed from Kupffer cells, localized around vessels in tumours and expressed key angiogenic markers, including VEGFA, TIE2, and CD34, signifying its essential role in tumour vascularization [73]. In addition, another pro-angiogenesis mechanism has been demonstrated in macrophages expressing lymphatic vessel endothelial hyaluronic acid receptor 1 (Lyve-1), residing proximal to blood vessels. James et al. uncovered that Lyve-1^+^ TAMs directly impacted αSMA^+^ CAF expansion via a PDGF-CC:PDGFRα interaction between these two populations to form a proangiogenic niche [74]. Additionally, the SPP1^+^ macrophage population exhibits proximal localization with fibroblasts in the TME to promote immunosuppression. Liu et al. demonstrated that SPP1^+^ macrophages located near the tumour border in HCC could interact with CAFs to stimulate extracellular matrix remodelling and tumour immune barrier formation [75]. Another study revealed that SPP1^+^ macrophages underwent reprogrammed functional states in the TME, highly expressing genes linked to inflammatory fibrosis, such as CTSB and LGALS3, and genes related to lipid metabolism, including APOE and APOC1, and this reprogrammed state of TAMs was induced by ligands derived from CAFs, including CSCF1, FGF1, PGF, TGFB3 and TIMP1 [60]. In addition, it has also been confirmed that SPP1 (also called osteopontin, OPN) expressed on macrophages contributes to T-cell activation via direct interaction with CD44 [76]. This evidence indicates that the localization of macrophages adjacent to CAFs implies the specific function of the macrophages, contributing to tumour growth and drug resistance. Moreover, it could be inferred that the spatial heterogeneity of TAMs is also an adaptive transformation to the tumour microenvironment. Carmona et al. found that TAMs in perivascular areas and hypoxic regions exhibited different phenotypes, with the former expressing mannose receptor C type 1 (MRC1) and the latter expressing arginase 1 (ARG1) [77]. In this study, it was proven that lactate, synergized with hypoxia, can induce the expression of ARG1 on TAMs via activation of MAPK signalling, and this phenotypic change was relevant to the angiogenic function of TAMs to restore blood perfusion.

In addition, TAM spatial heterogeneity also correlates with patient prognosis. C1Q^+^TREM2^+^APOE^+^ TAMs detected in tumour stroma were proven to correlate with cancer recurrence [28]. In another study, M1 and M2 macrophages were classified into seven predominant subgroups according to CD68, CD163, and CD206, in which the CD68^+^CD163^+^ TAMs localized in both the tumour nest and stroma, and a higher effective density of this subset in the tumour core correlated with better overall survival and relapse-free survival [78]. Analogously, Huang et al. investigated the spatial heterogeneity of TAMs in gastrointestinal Krukenberg tumours and unveiled that patients with high levels of infiltration of CD68^+^ TAMs and CD11c^+^ TAMs in the tumour nest of primary tumour tissues, instead of CD163^+^ TAMs, had worse overall survival and progression-free survival, and the infiltration level of CD68^+^ TAMs in the tumour nest of Krukrnberg tumours was related to overall survival as well, indicating the prognostic value of TAMs in the tumour nest [79]. Interestingly, contrary clinical outcomes could be associated with the same macrophage subset. A recent paper reported that CD169^+^ macrophages in primary tumours were associated with worse prognosis, while the CD169^+^ macrophages present in lymph node metastasis predicted a better prognosis, implying the protective role of CD169^+^ lymph node macrophages [80]. A similar result was reported by a previous study, in which the genes related to antigen presentation to B cells in CD169^+^ lymph node macrophages were upregulated, and their antimetastatic effects were B-cell acquisition, implying that this protective effect was B-cell-dependent [81].

## Macrophage–tumour stroma interactions facilitate immunosuppression

### Macrophage–fibroblast interactions in the tumour microenvironment

Fibroblasts constitute the most abundant stromal components of the TME, favouring immunosuppressive TME formation [82, 83]. CAFs are recognized as key regulators in tumour development by multiple mechanisms, comprising ECM remodelling, promotion of tumour cell proliferation and invasion, suppression of lymphocytes, and boosting angiogenesis [84–87]. In recent years, increased research has focused on CAFs and TAMs and discovered that their crosstalk is a significant motivation for tumour progression. Here, we summarize how macrophage–fibroblast interactions influence the functional states of each other and discuss what effect they generate in immune evasion and tumour development (Table 2).

**Table 2:** Macrophage-fibroblast interaction signalling in the tumour microenvironment

Macrophage-derived factors/receptors	Fibroblast-derived factors/receptors	Function	References
CSF1R	CSF1	Induce M2 polarization in monocytes	[88]
CCR2	CCL2	Promote myeloid cell infiltration	[90, 91]
unclear now	CXCL14	Increase macrophage infiltration	[92]
CXCR4	CXCL12	Promote macrophage recruitment and M2 polarization; promote LAM differentiation	[36, 93]
CXCR6	CXCL16	Promote macrophage recruitment	[95]
IL-6R	IL-6	Promote preinvasive TAM differentiation	
ST2	IL-33	Promote macrophage recruitment and polarization; promote MMP9 secretion of TAMs	[99]
C3aR	C3a	Promote monocyte differentiation to TAMs	[100, 101]
CXCL3	CXCR2	Induce CAF-to-myofibroblasts transition	[113]
OSM	OSMR	Induce inflammatory fibroblasts	[114]
IL-1β	IL-R1	Induce conversion of NFs to CAFs; Induce CAF production of IL-8 and GROα	[115, 116]
CXCR2	IL-8	Induce immunosuppressive TAMs to suppress NK cells	[102]
CXCR4	SDF-1	Induce immunosuppressive TAMs to suppress T cells	[107]
unclear now	HIF2	Promote macrophage M2 polarization; Regulate TAM expression of immune checkpoint	[109]
PTGIR	PGI_2_	Induce mixed-polarized TAM phenotype with reduced phagocytic capability	[125]
Granulin	unclear now	Activate myofibroblasts to induce ECM remodelling	[119]
TGF-β1	TGFβR2	Activate CAFs to induce ECM remodelling	[120]

#### Pro-tumour macrophage induction and immune suppression

**Fig. 2:**
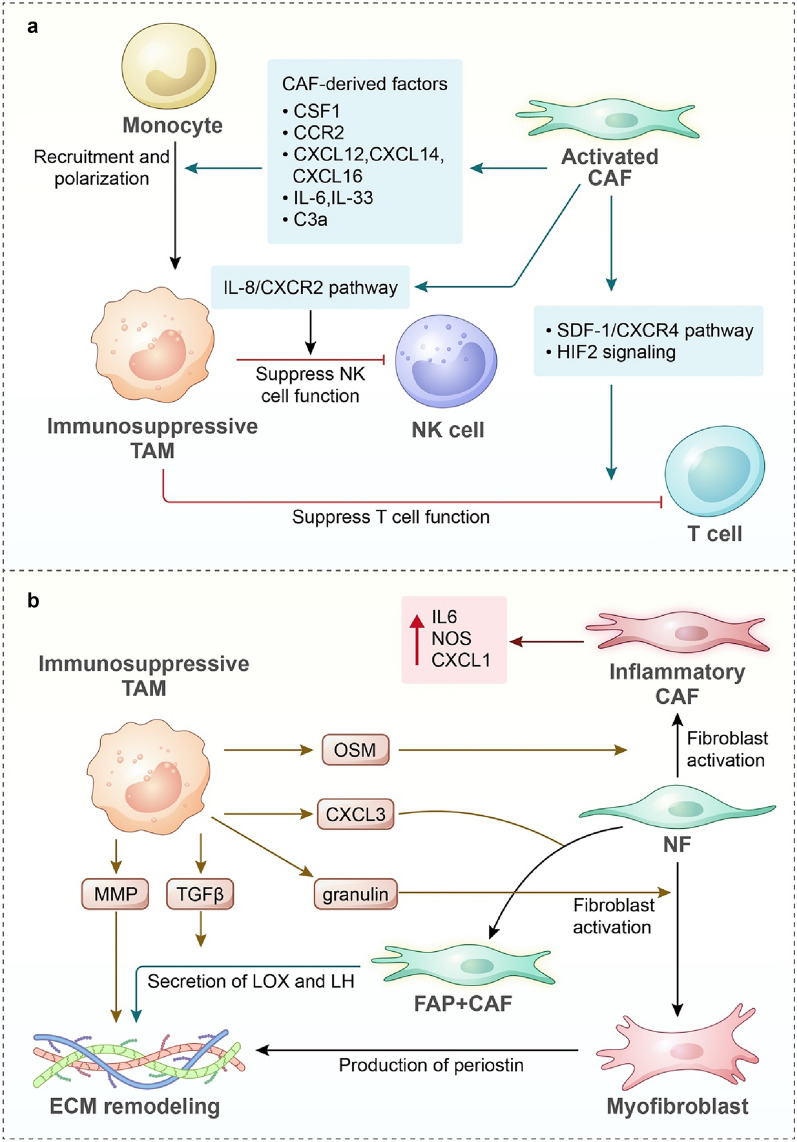
Macrophage–fibroblast interactions in the tumour microenvironment. **a** During tumour progression, activated CAFs (by cancer cells or TAMs) can secrete various cytokines to attract circulating monocytes towards the TME and then differentiate into TAMs. CAFs can suppress antitumour immunity by repressing both NK cells and T cells. **b** TAMs also secrete diverse factors to activate fibroblasts during immunosuppressive TME formation. Both TAMs and CAFs can remodel the tumour EMC. TAMs can secrete MMP to deposit collagen and promote fibrosis. TAM-activated CAFs can produce key enzymes related to EMC remodelling or directly produce ingredients

CAFs are capable of chemoattracting monocytes into the TME and regulating their differentiation (Fig. 2a). Emerging evidence has revealed that several chemokines are involved, including CSF1, CC chemokines, CXC chemokines, interleukins and complement components. The CSF1–CSF1R axis is an important signalling pathway in regulating TAM functions. CAFs can secrete CSF1 to stimulate ROS production in monocytes and subsequently lead to M2 polarization in the pancreatic cancer TME [88]. CCL2 synthesized by CAFs has been demonstrated to be a crucial factor in recruiting monocytes/macrophages towards the TME [89, 90]. Moreover, a recent study revealed that CAFs could induce CCL2 production in macrophages, thereby possibly attracting more monocytes towards the TME [91]. This evidence reveals a positive interaction feedback loop between CAFs and TAMs. In addition, CAFs are the source of CXCL12 and CXCL14, which can increase macrophage infiltration into TME [92, 93]. In this in vitro model, CXCL12 was the dominant chemoattractant to induce macrophage migration and M2 phenotype conversion, and these M2 TAMs could promote oral squamous cell carcinoma (OSCC) cell proliferation and CSC-like feature acquisition [93]. Similarly, Eleonora et al. revealed that inflammatory FAP^+^ CAFs could attract circulating myeloid cells to tumour sites via the CXCL12–CXCR4 pathway and induce macrophage differentiation towards LAM [36]. In HCC, CAF-derived CXCL12 could upregulate plasminogen activator inhibitor 1 in macrophages, eventually promoting tumour cell proliferation, migration and EMT [94]. Interestingly, Allaoui et al. found that monocytes in breast cancer could induce stroma formation and fibroblast activation, and then activated CAFs secreted CXCL16, which displayed a chemoattracting effect on monocytes [95]. Interleukins are another pivotal mediator of fibroblast–macrophage interactions. Previous studies have demonstrated that IL-6 is a pivotal cytokine that mediates CAF-cancer cell crosstalk and promotes cancer progression [96]. Recently, Higashino et al. confirmed that CAFs could induce macrophage recruitment and M2-type polarization by producing CCL2 and IL-6 [97]. In the research conducted by Cho et al., CAFs upregulated the secretion of IL-6 and GM-CSF in response to cancer cell stimulation, which cooperatively induced protumorigenic macrophages expressing M2 markers, including CD206, Arg1, and TGFβ [98]. IL-33 is another important regulatory cytokine that mediates CAF–TAM interactions, accounting for TAM recruitment and polarization. In pancreatic cancer models, CAF-secreted IL-33 commits macrophage M2 polarization [99]. Moreover, IL-33 could promote cancer metastases by stimulating MMP9 production in TAMs through ST2 receptors [99]. Furthermore, complement component C3a has been demonstrated to be an important mediator of macrophage differentiation [100]. Davidson et al. confirmed that C3a was produced most specifically by CAFs and were able to induce differentiation of recruited monocytes to macrophages [101].

Immune suppression is a hallmark of the TME, and fibroblast–macrophage interactions facilitate this process (Fig. 2a). Zhang et al. revealed that CAFs interacted with monocytes via the IL-8/CCR2 pathway, thereby inducing M2-type macrophages, which protected cancer cells from NK cell-mediated killing by reducing the cytotoxicity of NK cells [102]. Analogous results were reported by Louault et al. [103], in which they confirmed that the suppressive effect on NK cells from CAF-TAM collaboration relied on TGF-β1. TGF-β signalling is an important pathway involved in tumour immune evasion [104, 105]. A single-cell and spatial analysis revealed the interactions of SPP1^+^ macrophages and FAP^+^ fibroblasts in colorectal cancer mediated by TGF-β, IL-1, and chemerin, resulting in restricted T-cell infiltration [106]. Another study found that fibroblast–macrophage interactions were mediated by stromal cell-derived factor-1 (SDF-1, also called CXCL12), and CAF-educated TAMs markedly suppressed T-cell proliferation [107]. Macrophages are important mediators of the PD-1/PD-L1 axis in the TME, contributing to the suppression of the antitumour immune response. CAFs can produce hyaluronan to support the development of PD-L1^+^ macrophages from myeloid cells [108]. Additionally, PD-L1 expression in TAMs is controlled by CAF-derived hypoxia-inducible Factor 2 (HIF2) [109]. Zhu et al. discovered that a high level of PD-L1 expression in glioblastoma multiforme was positively correlated with M2-type TAM infiltration and negatively linked to the infiltration of CD8^+^ T-cells [110]. The mechanism is that PD-L1^+^ macrophages suppress the activity of CD8^+^ T-cells [111]. TAM-CAF crosstalk also induces PD-1 expression in macrophages [107], and this correlates negatively with the phagocytic potency of macrophages against tumour cells [112].

#### Fibroblast activation and extracellular matrix remodelling

Macrophage–fibroblast interactions also influence fibroblast activation and functions (Fig. 2b). Monocytes at primary tumours could induce fibroblasts expressing fibroblast activating protein (FAP) and promote fibroblast proliferation, indicating that the activation of CAFs was initiated by monocytes [95]. As mentioned above, IL-33 from CAFs could promote macrophage M2-type polarization [99]. Furthermore, Sun et al. discovered a feedback mechanism by which IL-33-stimulated macrophages could secrete CXCL3 to promote the phenotypic transformation of fibroblasts [113]. IL-33-activated M2 TAMs in pancreatic cancers led to high production of CXCL3 and conferred fibroblast-to-myofibroblast transformation via CXCL3-CXCR2 signalling. Myofibroblasts could promote cancer metastases through a mechanism by which cancer cells and myofibroblasts form clusters and co-metastases to distal tissues. Additionally, TAMs could interact with CAFs via secretion of oncostatin M (OSM) to reprogram fibroblasts, inducing an inflammatory fibroblast phenotype with increased expression of inflammatory mediator genes (Il6, Cxcl1, Nos, Il4ra) [114]. TAMs also regulate fibroblast activation by secreting interleukin. IL-1β from a mixed-polarized TAM subset could induce a conversion of noncancerous fibroblasts (NFs) to CAFs [115]. In addition, Young et al. reported that IL-1β was a key cytokine that mediated TAM-CAF crosstalk to promote inflammatory TME formation [116]. Macrophages in melanoma produce IL-1β and act on fibroblasts. IL-1β stimulated IL-8 and GROα production in fibroblasts, both of which further induced resistance to MAKP inhibition in melanoma cells. Intriguingly, some studies discovered that CAFs could be generated from macrophage–myofibroblast transition [117], implying complex relationships between these two TME components.

The extracellular matrix plays a vital and dynamic role in tumour progression due to its remodelling linked to lymphocyte infiltration in the TME and tumour cell metastases. CAFs are the key cellular components in ECM remodelling [118], and accumulating evidence shows that TAMs are also involved in this process via communication with CAFs (Fig. 2b). As mentioned before, a specific TAM subset, SPP1^+^ TAMs, colocalized with CAFs in the TME, contribute to the immune escape of tumour cells [60, 75, 106]. In-depth analysis revealed that SPP1^+^ macrophages crosstalk with CAFs via ligands, including TGFB1, SPP1, and IL1B, and these ligand‒receptor interactions resulted in the upregulation of ECM-related genes [75]. Sathe et al. discovered that other ligands from SPP1^+^ macrophages, including TNF, MMP9, and CCL2, could regulate target gene expression, including MMP2, VEGFA, and the collogen family, leading to fibrosis in metastatic colorectal cancer [60]. Moreover, CAFs also regulate the expression of SPP1 on TAMs via ligand‒receptor interactions (these CAF-derived ligands include CSF1, FGF1, PGF, TGFB3, and TIMP1) [60]. Overall, the signalling network between SPP1^+^ macrophages and CAFs influences the functional states of both cell types, promoting ECM remodelling in the TME. Furthermore, other studies confirmed multiple mechanisms of crosstalk between TAMs and CAFs inducing ECM remodelling. Nielsen et al. found that metastasis-associated macrophages secreted granulin to activate resident hepatic stellate cells into myofibroblasts, which produced periostin, an ECM component, inducing liver fibrosis to promote pancreatic cancer metastases [119]. Additionally, TAMs facilitate collagen crosslinking and stiffening by secreting TGF-β to stimulate the production of lysyl oxidases (LOX) and lysyl hydroxylases (LH) in CAFs [120]. Importantly, remodelled ECM facilitates tumour progression. Larsen et al. found that high collogen density, the hallmark of tumour tissues, instructed TAMs to acquire an immunosuppressive phenotype, which exhibited inefficient potency in attracting cytotoxic T cells and an enhanced capability to inhibit T-cell proliferation [121]. In addition, ECM remodelling induces the generation of a desmoplastic microenvironment that prevents lymphocyte infiltration [106].

Additionally, recent research has discovered that CAFs also directly involved in tumor vascularization by secreting pro-angiogenic factors, such as VEGF and SDF-1, or remodelling ECM to favor vessel formation [122, 123]. It also has been demonstrated that M2 type TAMs play important role in angiogenesis, which would be introduced later. Since CAFs could induce M2 phenotypic transmission in TAMs, one wonders whether CAFs may indirectly promote angiogenesis by affecting the proangiogenic capacity of TAMs. However, the interplay among CAFs, TAMs and endothelial cells remains unclear. Up to now, there is still a lack of evidence directly demonstrating the pro-angiogenic capacity of TAMs induced by CAFs. Lately, Luo et.al. found a subpopulation of CAFs exhibited a pro-angiogenic hallmark, originating from endothelial cells by endothelial–mesenchymal transition (EndMT) [124]. And SPP1^+^ TAMs promoted this phenomenon via SPP1–CD44 interaction. Although this result couldn’t replay our question, it confirmed crosstalk among CAFs, TAMs and endothelial cells facilitated tumor vascularization. Thus, we speculate that both CAFs and TAMs could modulate each other’s phenotypes and functions to promote tumor angiogenesis, but more in-depth exploration is demanded for direct evidence in future.

### Intersection between angiogenesis and immunosuppression in the tumour microenvironment

#### Macrophage-endothelia crosstalk

**Fig. 3:**
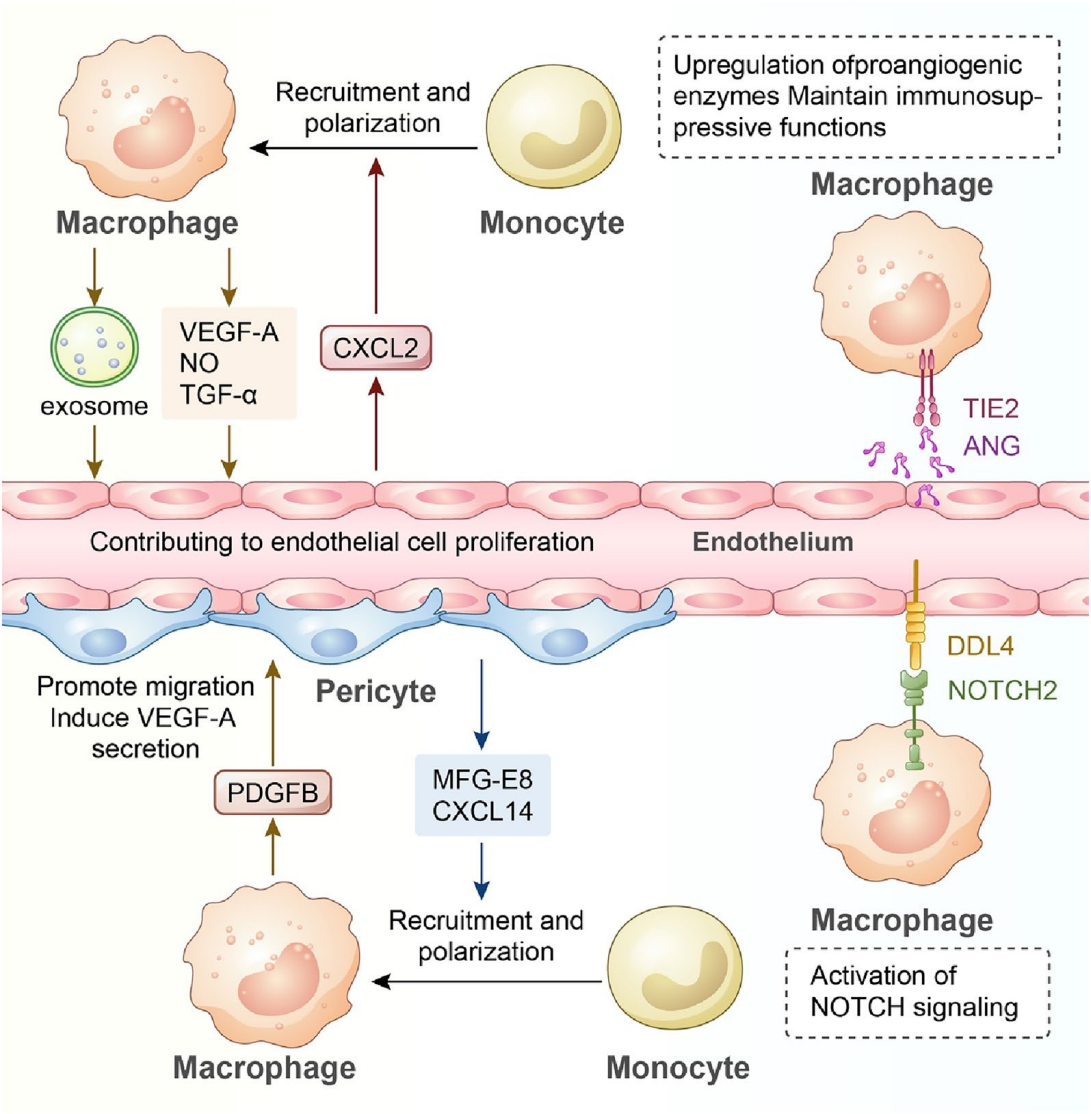
Macrophages interact with ECs or pericytes during tumour progression. TAM–EC interactions favour tumour angiogenesis: (1) TAMs secrete a multitude of cytokines or exosomes to activate ECs and enhance EC proliferation; (2) the ANG2-TIE2 axis is involved in angiogenesis by upregulating proangiogenic enzyme expression in TAMs. TAM–EC interactions in turn regulate TAM functions. ECs secrete CXCL2 to promote TAM recruitment. ANG2 from ECs also induces immunosuppressive TAMs in the TME. The TAM-EC crosstalk mediated by the DDL4:NOTCH2 ligand‒receptor interaction activates NOTCH signalling in TAMs, contributing to the formation of an immunosuppressive niche. Pericytes are involved in TAM recruitment and polarization via the production of MFG-E8 and CXCL14. TAMs induce pericyte migration and proangiogenic activity via PDGFB-PDGFRβ signalling

Tumour angiogenesis and immunosuppression are not irrelevant pathological processes but potently affect each other. Numerous studies have reported that TAMs localized in the perivascular niche can facilitate tumour angiogenesis (Fig. 3). It has been demonstrated that TAMs are able to secrete VEGF-A to activate ECs and initiate vascularization [58, 77]. The angiopoietin-2 (ANG2)-TIE2 axis is also a crucial mechanism for tumour angiogenesis. Hughes et al. discovered that proangiogenic MRC1^+^TIE2^+^ TAMs were associated with tumour revascularization and relapse [126]. Blockade of ANG2 impeded upregulation of Tie2 in MRC1^+^TIE2^+^ TAMs, impairing their proangiogenic capability [127]. While overexpression of ANG2 increased the infiltration of TIE2^+^ TAMs and upregulated their expression of proangiogenic enzymes (thymidine phosphorylase and cathepsin B), IL-10, MRC1, and CCL17 [128]. Apart from the ANG2-dependent pathway, perivascular TAMs could secrete NO and TNF to activate ECs in lung metastasis of breast cancers, forming a vascular niche favouring tumour metastasis [129]. TNFα from TAMs can induce increased expression of genes in ECs, including VCAM-1, ICAM-1, CXCL5, and CXCL10, and this EC activation pathway may contribute to resistance to anti-VEGF therapy [130]. It also reported that crosstalk between TAMs and tumour endothelial cells is mediated by exosomes, which were able to transport exosomal miRNAs into ECs to downregulate the expression of the transcription Factor E2F2, thereby promoting EC proliferation [131]. Mutually, angiogenesis also participates in modulating the tumour immune microenvironment (Fig. 3). Proangiogenic factors can negatively affect antigen-presenting cells and effector T cells but augment immunosuppressive cells, including Tregs, TAMs, and myeloid-derived suppressor cells (MDSCs) [132, 133]. Moreover, the ANG2-TIE2 axis also regulates macrophage functions in the TME to facilitate immune evasion. IL-10 from ANG2-induced TAMs could suppress T-cell proliferation and promote the expansion of suppressive FOXP3^+^ Tregs [134]. In these TIE2-expressing macrophages, the activities of TIE2 and VEGFR kinase maintain a high level of costimulatory ligand CD86, contributing to the conversion of T cells to Tregs [135]. Recently, a single-cell study reported that foetal-associated PLVAP^+^ endothelial cells and foetal-like FOLR2^+^ macrophages colocalized in the liver tumour ecosystem to form an immunosuppressive niche, in which PLVAP^+^ ECs activate Notch signalling in macrophages via specific ligand‒receptor interactions (DLL4:NOTCH2) to promote TAM reprogramming [48]. In addition, the EC-derived cytokine CXCL2 could drive macrophage recruitment and induce an immunosuppressive phenotype to suppress T-cell cytotoxicity [136].

#### Communication between macrophages and pericytes

Pericytes, described as cells constituting the basic structure of capillaries together with ECs, basement membrane, and vascular smooth muscle cells, play important roles in tumour angiogenesis. TAMs promote tumour angiogenesis by relying on communication with not only ECs but also pericytes (Fig. 3). In an in vitro assay, triple coculture of macrophages, ECs, and pericytes had a multiplicative effect on blood vessel sprouting [137]. It is well known that TAM-pericyte crosstalk via PDGFB–PDGFRβ signalling can enhance tumour angiogenesis. In malignant glioma, CECR1 regulates PDGFB production in M2 TAMs, and TAM-derived PDGFB could promote PDGFRβ^+^ pericyte migration and secretion of VEGF-A and the proangiogenic ECM component periostin, thereby contributing to new vessel formation [138]. There is bidirectional dialogue between TAMs and pericytes. TAMs promote pericyte recruitment and activation, and pericytes are involved in TAM recruitment and polarization. Previous studies reported that milk fat globule EGF Factor 8 (MFG-E8) from pericytes and mesenchymal stem cells is associated with macrophage M2 polarization in melanoma [139]. Recently, Wang et al. found that fibroblast growth factor-2 (FGF-2) from nasopharyngeal carcinoma strongly promoted the expression of CXCL14 in pericytes via FGFR1/AHR signalling, which promoted TAM infiltration and M2 polarization [140]. Meanwhile, it has also been reported that TAMs are the primary source of FGF-2 in the TME, and FGF-2 can directly interact with its receptor FGFR1 on TAMs to induce a protumour phenotype [141]. However, current research focusing on TAM-pericyte crosstalk remains insufficient, and whether the above mechanisms are universally applicable in multiple tumours or whether other signalling pathways mediate their communication is worth further investigation.

### Macrophage–mesenchymal stem cell interactions in the immunosuppressive TME

**Fig. 4:**
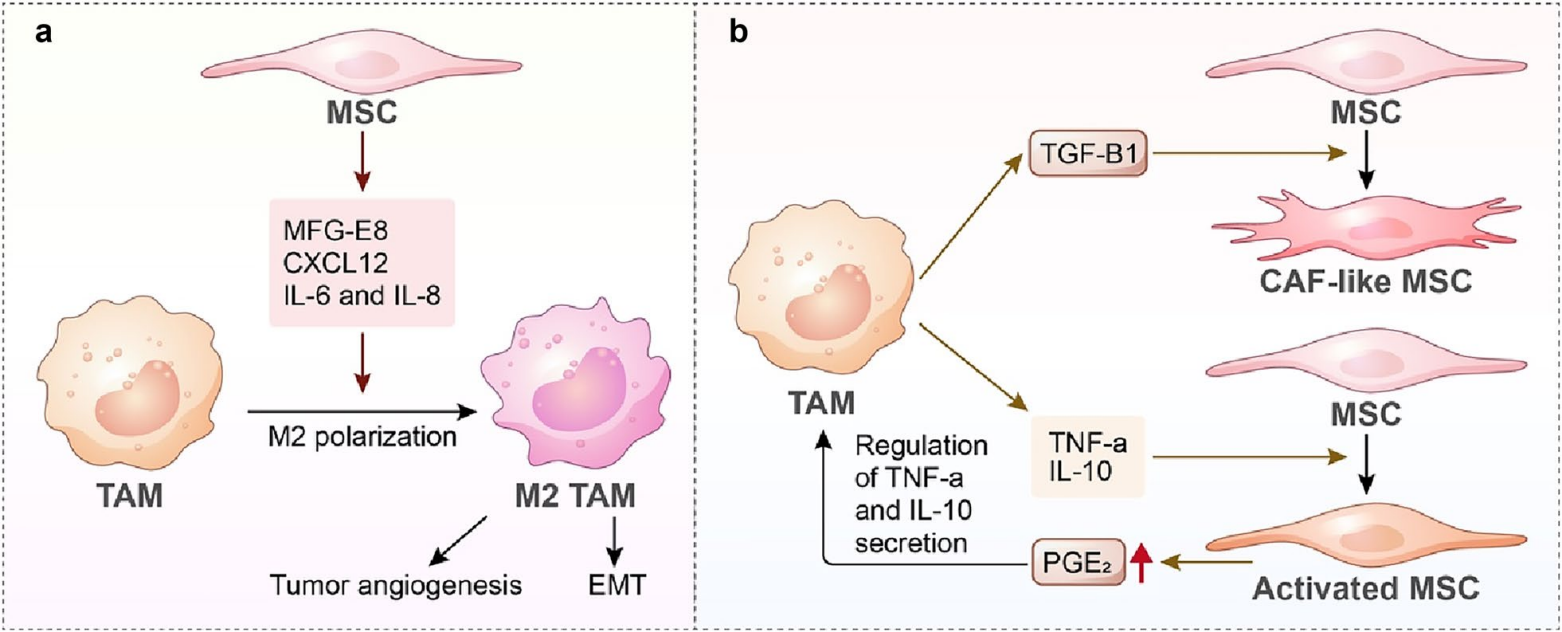
TAM–MSC interactions in the tumour microenvironment. **a** MSCs promote M2 polarization of macrophages via cytokines, including MFG-E8, CXCL12, IL-6, and IL-8. MSC-primed M2 TAMs promote tumour angiogenesis and EMT. **b** TGF-β1 from TAMs induces MSC differentiation into CAF-like MSCs. TNF-α and IL-10 from TAMs are priming factors of MSCs, which in turn upregulate the expression of PGE_2_ to regulate the immunomodulatory potential of TAMs

MSCs, known as multipotent mesenchymal stromal cells, which retain differentiation capability and stromal surface markers, are key protumor components in the TME. Emerging evidence suggests that MSCs exert immunosuppressive potential within the TME by interacting with several immune cells, such as macrophages (Fig. 4). As discussed above, MFG-E8 from MSCs drives melanoma progression by stimulating macrophage M2 polarization, thus contributing to tumour angiogenesis [139]. Recent studies discovered that other MSC-derived cytokines promote macrophage polarization. In a breast cancer model, CXCL12 produced by MSCs could switch the TAM phenotype to M2 [142]. In gastric cancer, Li et al. confirmed that MSCs contributed to TAM M2 polarization by secreting IL-6 and IL-8, and these MSC-primed TAMs could subsequently prompt tumour cell EMT [143]. Interestingly, in a leukaemia mouse model under MSC treatment, donor MSCs could reprogram TAMs to execute tissue-repairing functions, suppressing leukaemia progression [144]. In addition, Zheng et al. reported that bone marrow-derived MSCs injected at sites distant from tumours could decrease the infiltration of myeloid-derived suppressor cells and Tregs within tumours and enhance antitumour immunity [145]. These results indicate that MSCs are heterogeneous populations in the TME with both protumor and antitumour capabilities. Similar to interactions between TAMs and other tumour stromal cells, TAM–MSC crosstalk is bidirectional, which means that the phenotypes of TAMs can be regulated by MSCs within the TME, while the activation or transition of MSCs is also affected by TAMs in turn. In vitro, TNF-α and IL-10 from macrophages could prime MSCs, which in turn exert enhanced immunomodulatory effects on macrophages by PGE_2_ secretion [146]. Furthermore, TGF-β1 was proven to be a crucial cytokine mediating TAM–MSC crosstalk, which could recruit MSCs and induce MSC differentiation into a CAF-like phenotype [147].

### Macrophages in cancer-nerve intersection

**Fig. 5:**
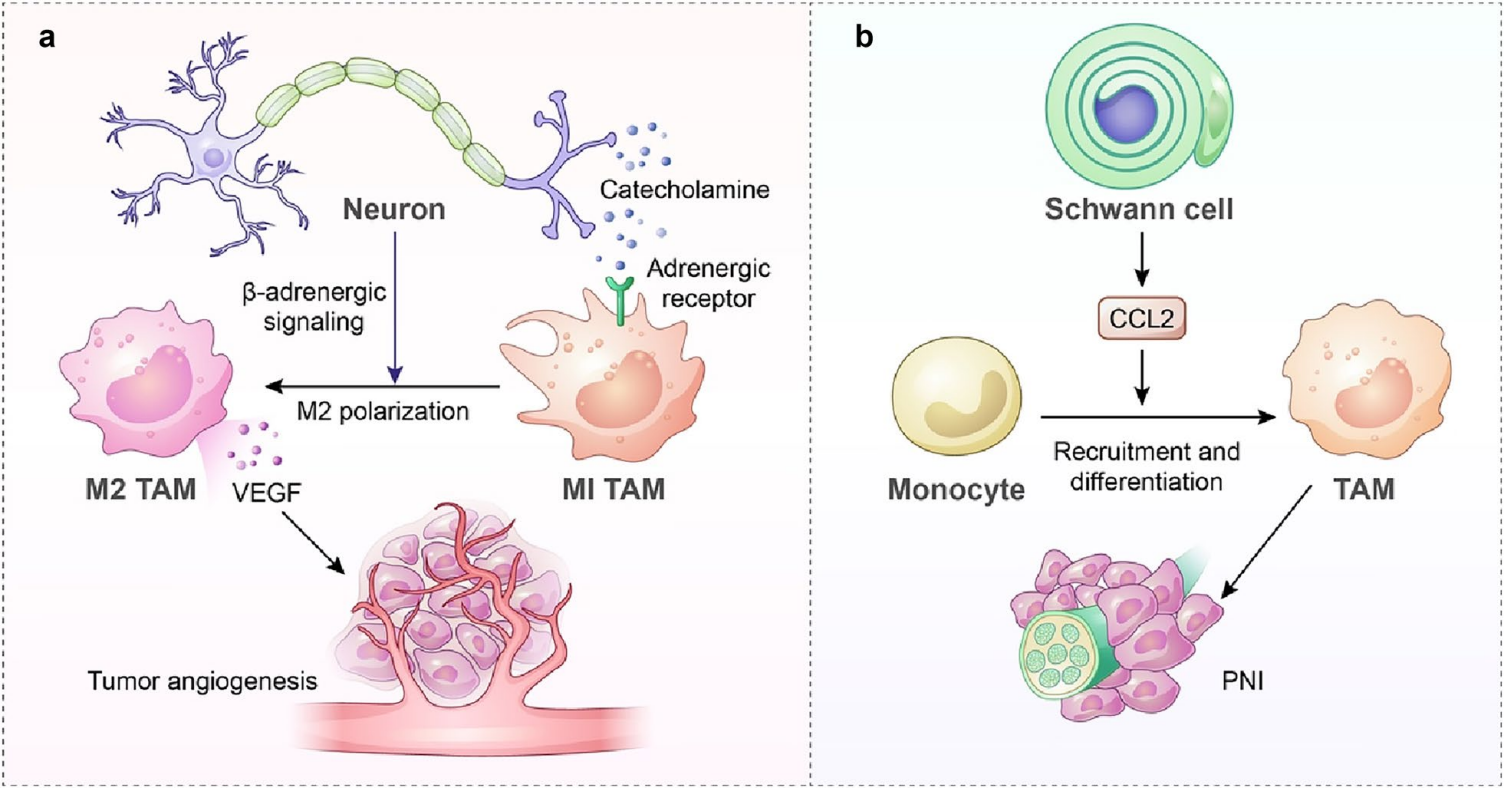
Macrophages are involved in neuro-immune interactions to promote tumour progression. **a** Neurons induce M2 polarization and VEGF secretion of TAMs mediated by β-adrenergic signalling, contributing to tumour angiogenesis. **b** Monocytes are recruited towards TME CL2 cells derived from Schwann cells and differentiate into TAMs to promote nerve invasion

Nervous cells have been regarded as essential components of the TME. Communication between the nervous and immune systems has undergone extensive exploration. Neurons can promote immunosuppression by inducing T-cell exhaustion by β-adrenergic signalling or neuropeptides [148, 149]. Neurons also facilitate tumour-promoting inflammation via direct or indirect interactions with macrophages (Fig. 5). It has been reported that neurons in low-grade gliomas can produce midkine to activate CD8^+^ T-cells, which in turn increases CCL4 secretion to induce the expression of CCL5 in microglia, thereby favouring tumour cell survival [150]. In addition, neurons in the TME also regulate macrophage recruitment by releasing neurotransmitters. Sloan et al. observed that the sympathetic nervous system could promote the infiltration of F4/80^+^ macrophages into tumour sites and induce an M2 phenotype mediated by β-adrenergic signalling [151]. Further investigation proved that these adrenergic-stimulated TAMs can promote tumour angiogenesis by secreting VEGF [152]. Moreover, nerve–macrophage interactions also induce tumour metastasis by perineural invasion (PNI). PNI is defined as a pathologic process in which tumour metastasis is mediated by malignant cell-invaded nerves [153]. Schwann cells can secrete CCL2 to chemoattract CCR2-expressing monocytes to the PNI region, where they differentiate into macrophages to enhance nerve invasion in a cathepsin B-dependent manner [154].

Currently, the interplay between immune cells and neurons has attracted great attention, and the definition of “neuroimmune cell units” has been proposed to describe the anatomical location where immune and nervous cells colocalize and functionally interact to steer tissue physiology and protection [155]. Neuron-macrophage interactions in the healthy stage for intestinal motility and anti-infection defence have been investigated extensively [156]. However, studies focusing on macrophage–neural cell interactions in the TME are quite limited, and further investigation is needed.

## Targeting TAMs for tumour therapy

Current evidence confirms that macrophages are the main inducers responsible for immunosuppression in the TME. There is considerable therapeutic potential in targeting TAMs to synergize with cancer immunotherapy. Strategies for targeting macrophages can be roughly divided into the following two aspects: (1) decreasing immunosuppressive TAMs by either eliminating those already present in the TME or inhibiting their recruitment and (2) reversing their immunosuppressive phenotype. Notably, the TAM–CAF interaction pathway is also a promising therapeutic target for enhancing immunotherapy. Currently, chimeric antigen receptor (CAR) macrophages have become an alternative approach for cancer immunotherapy. An increasing number of studies have reported the excellent antitumour effects of CAR macrophages in animal models. Here, we summarize current preclinical or clinical advancements in TAM-targeted therapy, and in particular, we focus on recent advancements in CAR macrophage therapy.

### Therapies for decreasing immunosuppressive TAMs

A main strategy to deplete TAMs is disrupting signalling associated with myeloid cell recruitment and differentiation to macrophages. Since TAM–CAF interactions play crucial roles in TAM recruitment and polarization, blocking their crosstalk is a promising approach. Many CAF-derived cytokines, such as CSF-1, have been proven to be involved in regulating macrophage migration and activation. Activation of CSF-1/CSF-1R signalling can promote macrophage accumulation and polarization in the TME [157]. Although blockade of this pathway cannot directly lead to tumour cell elimination, it provides improved efficacy of immunotherapy due to significantly decreased immunosuppressive TAMs. Zhu et al. reported that hindering CSF-1 signalling with CSF-1R inhibitors elicited a reduction in TAMs and an increase in CD8^+^ T-cell infiltration, sensitizing an HCC mouse model to anti-PD-L1 therapy [158]. To date, diverse drugs targeting CSF-1/CSF-1R signalling have been tested for clinical traits, including antagonistic monoclonal antibodies and small-molecule inhibitors. Emactuzumab, an anti-CSF1R antibody, showed marked effectiveness as a single agent in patients with giant cell tumours [159]. Recently, emactuzumab combined with anti-PD-L1 atezolizumab has been evaluated in a phase Ib study, showing increased therapeutic benefits [160]. Other CAF-derived cytokines that mediate TAM-CAF crosstalk include CC chemokines and CXC chemokines. CAF-derived CCL2 binds to its receptor CCR2, evoking monocyte recruitment and differentiation into TAMs. Li et al. utilized a CCR2 antagonist in HCC models and observed repressed M2-type TAM infiltration and an activated CD8^+^ T-cell immune response [161]. CXCL12-CXCR4 signalling also mediates crosstalk between macrophages and fibroblasts in TAMs, which is associated with TAM recruitment and polarization. It has been demonstrated that CXCR4 inhibition could counter immunosuppression resulting from increased hypoxia after sorafenib treatment by reducing the infiltration of immunosuppressive cells, such as M2 TAMs and Tregs [162]. By suppressing TAM recruitment to reprogram the TME towards antitumour activity, CXCR4 antagonists show synergistic effects in combination with anti-PD-1 therapy [163]. In addition, IL-33 is another key interaction mediator between CAFs and macrophages. By knocking out ST2, the IL-33 receptor, in mouse CRC models to disrupt IL-33/ST2 signalling, an enhanced CD8^+^ T-cell immune response was observed, indicating that ST2 is a potential checkpoint target for immunotherapy [164].

The clearance of immunosuppressive TAMs already present in the TME is another option to alleviate immunotherapy resistance. A feasible method might be employing cytotoxic T cells to deplete TAMs. CAR-T-cell therapy, which can specifically recognize and eliminate cells expressing specific antigens, has been acknowledged as a revolutionary cancer therapy [165]. Garcia et al. developed a CAR-T-cell platform to selectively eliminate M2 macrophages expressing folate receptor β and confirmed that the platform could significantly decrease immunosuppressive M2-type TAMs and increase proinflammatory monocytes within tumours, eliciting delayed tumour growth and TME reshaping [166]. Unfortunately, insufficient infiltration of engineered T cells into solid tumours limits their therapeutic effect and acts as a possible barrier for this approach.

### Targeting TAMs for phenotypic reprogramming

Another strategy underlies the reprogramming of macrophages from anti-inflammatory to proinflammatory, thereby favouring an antitumour immune response. Triggering receptor expressed on myeloid cells-2 (TREM2)-mediated signalling is highly related to macrophage functions, including phagocytosis and inflammation regulation [167]. In multiple tumours, TREM2 expression is negatively correlated with the infiltration of immune cells, including dendritic cells, lymphocytes, and NK cells [168]. A single-cell study demonstrated that TREM2^+^ macrophages within tumours suppressed CD8^+^ T-cell functions, contributing to tumour immune escape [38]. In a preclinical study, Binnewies et al. discovered that anti-TREM2 monoclonal antibody treatment induced profound changes in the macrophage compartment, including a reduction in M2-type TAMs and an increase in the proportion of TAMs expressing proinflammatory genes [169]. Moreover, anti-TREM2 also increases INFγ and TNFα production by intertumoral CD4^+^ and CD8^+^ T cells in sarcoma models and enhances anti-PD-L1 immunotherapy [170]. Thus, targeting macrophages to reprogram their phenotypes is a feasible and potent approach to reshape the immunosuppressive TME, favouring immunotherapy. CD47, a ligand expressed on tumour cells, has also attracted great attention because its interaction with the macrophage receptor SIRPα can regulate macrophage phagocytic capability. In breast cancer models, anti-CD47 monoclonal antibodies combined with targeting-STING therapy promoted macrophage phagocytosis of tumour cells and CD8^+^ T-cell priming [171]. In terms of clinical traits, blockade of CD47 combined with rituximab showed considerable effectiveness for patients with non-Hodgkin’s lymphoma [172].

Importantly, activated signalling linked with the antitumour activity of macrophages can repolarize TAMs, for example, CD40 signalling. CD40 is a costimulatory molecule expressed by some macrophages, whose activation can stimulate IL-12 production and induce a T-cell-mediated immune response [173]. Liu et al. confirmed that CD40 signalling induced macrophage metabolic reprogramming and controlled the activation of proinflammatory and antitumorigenic polarization [67]. In pancreatic carcinoma models, agonistic CD40 monoclonal antibodies induce upregulation of MHC-II and costimulatory molecule CD86 expression on TAMs, driving the restoration of immune surveillance [174]. Recently, Weiss et al. applied sotigalimab, a CD40 agonist, combined with a CSF1R inhibitor in NSCLC patients with anti-PD-1/PD-L1 resistance, achieving effective TME reshaping and antitumour activity [175].

### Engineered macrophages for immunotherapy

CAR-T therapy is considered a revolutionary technique for cancer immunotherapy. However, as mentioned before, low infiltration of T cells into tumours is a major hurdle, and it has also been reported that an immunosuppressive TME limits treatment efficacy [176]. Macrophages account for up to 50% of infiltrating cells at tumour sites and can infiltrate tumours more efficiently than T cells. Recently, CAR macrophages have emerged as an alternative approach. Klichinsky et al. constructed CD3ζ-based anti-HER2 CAR macrophages for cancer immunotherapy by reversing the suppressive immune microenvironment [177]. In this study, it was demonstrated that anti-HER2 CAR macrophages induced a proinflammatory TME. Anti-HER2 CAR macrophages could directly phagocytose HER2^+^ tumour cells. Meanwhile, they upregulated the expression of costimulatory ligands and cross-presented tumour antigens to activate T cells. This finding implies that CAR macrophage therapy is an effective approach for overcoming immunosuppression and evoking antitumour immunity in the TME. As discussed before, ECM remodelling in the TME is a key factor that restricts lymphocyte infiltration. A CAR-147 macrophage targeting tumour ECM for alleviation of immune suppression was generated by Zhang et al. [178]. The CAR-147 macrophages were capable of binding to HER2^+^ cancer cells, leading to intercellular CD147 activation and thereby upregulating the expression of MMPs. Consequently, CAR-147 macrophages can promote T-cell infiltration into solid tumours by reducing tumour ECM deposition.

However, the clinical translation of CAR macrophage therapy poses some challenges. For example, bone marrow or peripheral blood mononuclear cell-derived macrophages are not efficiently engineered, and CAR macrophages cannot proliferate either ex or in vivo [179, 180]. Recently, pluripotent stem cells (iPSCs) were used to induce iPSC-derived CAR macrophages to eliminate cancer cells [180]. The CAR was first transduced into iPSCs, and then the CAR-iPSCs were induced to differentiate towards macrophages. The iPSC-derived CAR macrophages showed antigen-dependent antitumour functions, including M1-type polarization, cytokine secretion, enhanced phagocytosis of tumour cells, and significant anticancer activity in vivo. In addition, reprogramming macrophages towards CAR macrophages in vivo via a nanodrug delivery system has been reported. Kang et al. synthesized nanocomplexes containing the CAR-IFN-γ gene, which could program M2 TAMs towards CAR-M1 macrophages in the TME [181]. These CAR-M1 macrophages exert strong immunomodulating effects, being able to activate CD8^+^ T-cells and decrease Treg infiltration and the expression of anti-inflammatory cytokines. In another study, CAR gene-containing nanocarriers were embedded in injectable hydrogels, which also induced CAR macrophages and shifted their phenotype from M2 to M1, thus resulting in activation of the adaptive immune response [182]. CAR macrophage therapy has shown great potential in cancer immunotherapy, but to date, only two CAR macrophage-based clinical trials have been approved by the FDA (NCT03608610 and NCT04660929). More clinical research is needed in the future to demonstrate the safety and effectiveness of CAR macrophage therapy.

## Discussion and conclusion

Macrophages are essential cellular components in host innate immunity and have an indispensable role in boosting the inflammatory response but lose their protective functions in the context of cancer. Lately single-cell studies have concentrated on TAMs and revealed they participated in multiple biological process, including carcinogenesis, invasion, and metastasis. Collectively, we summarized research advancement on TAMs and their complex interplay networks. Our findings could be condensed in following 3 key points: (1) TAM heterogeneity, (2) TAMs–stromal cell interactions, and (3) TAM-targeted therapy.

In detail, TAMs are heterogeneous and plastic populations within tumours. TAMs can be divided into various clusters with different metabolic features, phenotypes, and spatial distributions, which are highly correlated with their functional states. Numerous TAM subsets show altered metabolic properties (enhanced lipid metabolism and aerobic glycolysis) and phenotypes with immunosuppressive hallmark during tumour progression. Lately, increasing studies have concentrated on crosstalk between macrophage and tumour stromal cells in TME. We hold a point of view that non-cancerous cells in TME are key determinants of solid tumour characteristics. In this review, we have presented how TAMs and stromal cells influence tumour biological behaviours. These non-cancerous cells communicated via soluble cytokines or direct ligand-to-receptor interactions to regulate each other’s transcriptional profiling and subsequent functional states. And then affect the tumour property and patient prognosis. For example, TAM–CAF interactions promote ECM remodelling, thereby restraining T cells infiltration into cancer nest [75]. This effect boosts immunosuppression and then restricts efficacy of immune checkpoint inhibitors. Among these cells, we believe that macrophage located at the central position in the interplay networks, due to that TAMs participated in activation of stromal cells, regulated their functions, and cooperated to perform pro-tumour effect. Hence, targeting TAMs might be an effective and promising strategy for cancer immunotherapy. Nowadays, strategies for targeting TAMs comprise reducing or reprogramming pro-tumour macrophages. Multitude studies have demonstrated that both approaches are effective for arousing antitumour immunity, synergizing with conventional immunotherapy, such as anti-PD-1 therapy. In recent years, engineered cells have shown great potential in oncotherapy. Accumulating studies have reported that CAR macrophage therapy could reverse immunosuppression in the TME, indicating that it is a promising direction for future immunotherapy.

Currently, exploration of TME has become a scientific hot point in oncology. The TAM has become the star cell in this field and quantities of research articles published every year, revealing its key role in influencing biological behaviors of tumor cells, regulating tumor stromal microenvironment, and inducing immune escape. However, there are still issues required future research. As we introduced above, most studies revealed monocyte-derived TAMs interacted with CAFs or other stromal cells to remodel TME. What about TRM? Some research has proved TRMs also involved in tumor progression, especially in lung pre-metastasis niche [183, 184], but few studies concentrated on their interactions with stromal cells. Exploring TRMs and their cellular communications in TME may contribute to understanding macrophage heterogeneity and cell crosstalk. As for TAM-targeted therapy, more research is needed for improved efficacy. Based on our understanding of TAM heterogeneity, since pro-tumour macrophages exhibit altered metabolic features, specific phenotypes, and different spatial distributions, targeting these properties of TAMs may be a prospect for oncotherapy. Importantly, macrophage–stroma interactions contribute to immunosuppressive TME formation and tumour progression. Thus, obstructing this crosstalk can reverse this suppressive status, promising enhanced immunotherapy efficacy.

## Data Availability

Not applicable.

## Abbreviations

TME: Tumor microenvironment
TAM: Tumour-associated macrophage
ECM: Extracellular matrix
NK cell: Natural killer cells
CAF: Cancer-associated fibroblasts
EC: Endothelial cell
MSC: Mesenchymal stem cell
TLR: Toll-like receptor
IFN: Interferon
TNF: Tumour necrosis factor
IL: Interleukin
TRM: Tissue-resident macrophage
PDPN: Podoplanin
LAM: Lipid-associated macrophage
Treg: Regulatory T lymphocyte
EMT: Epithalami–mesenchymal transition
PPARγ: Peroxisome proliferator-activated receptor γ
MMP: Matrix metalloprotease
BC: Breast cancer
HCC: Hepatocellular carcinoma
ccRCC: Clear cell renal carcinoma
NSCLC: Non-small cell lung cancer
GBM: Glioblastoma
MEL: Melanoma
PDAC: Pancreatic ductal adenocarcinoma
LCA: Lung cancer
SCC: Squamous cell carcinoma
CRC: Colorectal cancer
ATC: Anaplastic thyroid cancer
EMT: Epithelial–mesenchymal transition
PAR1: Protease-activated receptor 1
MHC: Major histocompatibility complex
Lyve-1: Lymphatic vessel endothelial hyaluronic acid receptor 1
MRC1: Mannose receptor C type 1
ARG1: Arginase 1
TGF: Transforming growth factor
OSM: Oncostatin M
NF: Noncancerous fibroblast
LOX: Lysyl oxidase
LH: Lysyl hydroxylase
ANG2: Angiopoietin-2
MDSC: Myeloid-derived suppressor cell
FGF-2: Fibroblast growth factor-2
PNI: Perineural invasion
CAR: Chimeric antigen receptor
iPSC: Pluripotent stem cell
